# Guidance for conducting feasibility and pilot studies for implementation trials

**DOI:** 10.1186/s40814-020-00634-w

**Published:** 2020-10-31

**Authors:** Nicole Pearson, Patti-Jean Naylor, Maureen C. Ashe, Maria Fernandez, Sze Lin Yoong, Luke Wolfenden

**Affiliations:** 1 School of Medicine and Public Health, University of Newcastle, University Drive, Callaghan, NSW 2308 Australia; 2 Hunter New England Population Health, Locked Bag 10, Wallsend, NSW 2287 Australia; 3 School of Exercise Science, Physical and Health Education, Faculty of Education, University of Victoria, PO Box 3015 STN CSC, Victoria, BC V8W 3P1 Canada; 4 Center for Health Promotion and Prevention Research, University of Texas Health Science Center at Houston School of Public Health, Houston, TX 77204 USA; 5 Department of Family Practice, University of British Columbia (UBC) and Centre for Hip Health and Mobility, University Boulevard, Vancouver, BC V6T 1Z3 Canada

**Keywords:** Feasibility, Pilot, Hybrid trial designs, Implementation science

## Abstract

**Background:**

Implementation trials aim to test the effects of implementation strategies on the adoption, integration or uptake of an evidence-based intervention within organisations or settings. Feasibility and pilot studies can assist with building and testing effective implementation strategies by helping to address uncertainties around design and methods, assessing potential implementation strategy effects and identifying potential causal mechanisms. This paper aims to provide broad guidance for the conduct of feasibility and pilot studies for implementation trials.

**Methods:**

We convened a group with a mutual interest in the use of feasibility and pilot trials in implementation science including implementation and behavioural science experts and public health researchers. We conducted a literature review to identify existing recommendations for feasibility and pilot studies, as well as publications describing formative processes for implementation trials. In the absence of previous explicit guidance for the conduct of feasibility or pilot implementation trials specifically, we used the effectiveness-implementation hybrid trial design typology proposed by Curran and colleagues as a framework for conceptualising the application of feasibility and pilot testing of implementation interventions. We discuss and offer guidance regarding the aims, methods, design, measures, progression criteria and reporting for implementation feasibility and pilot studies.

**Conclusions:**

This paper provides a resource for those undertaking preliminary work to enrich and inform larger scale implementation trials.

## Background

**Fig. 1:**
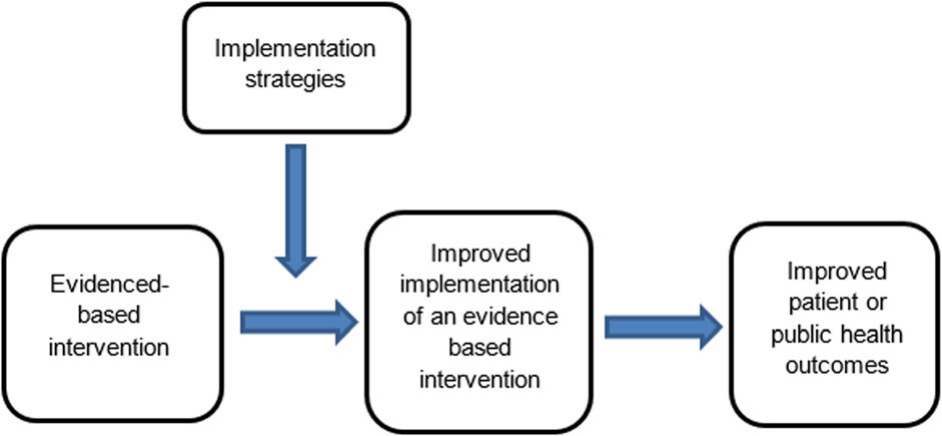
Conceptual role of implementation strategies in improving intervention implementation and patient and public health outcomes

The failure to translate effective interventions for improving population and patient outcomes into policy and routine health service practice denies the community the benefits of investment in such research [1]. Improving the implementation of effective interventions has therefore been identified as a priority of health systems and research agencies internationally [2–6]. The increased emphasis on research translation has resulted in the rapid emergence of implementation science as a scientific discipline, with the goal of integrating effective medical and public health interventions into health care systems, policies and practice [1]. Implementation research aims to do this via the generation of new knowledge, including the evaluation of the effectiveness of implementation strategies [7]. The term “implementation strategies” is used to describe the methods or techniques (e.g. training, performance feedback, communities of practice) used to enhance the adoption, implementation and/or sustainability of evidence-based interventions (Fig. 1) [8, 9].

**Table:** 

**Definitions**	
Feasibility studies: an umbrella term used to describe any type of study relating to the preparation for a main study	
*Pilot studies*: a subset of feasibility studies that specifically look at a design feature proposed for the main trial, whether in part or in full, conducted on a smaller scale [10]

While there has been a rapid increase in the number of implementation trials over the past decade, the quality of trials has been criticised, and the effects of the strategies for such trials on implementation, patient or public health outcomes have been modest [11–13]. To improve the likelihood of impact, factors that may impede intervention implementation should be considered during intervention development and across each phase of the research translation process [2]. Feasibility and pilot studies play an important role in improving the conduct and quality of a definitive randomised controlled trial (RCT) for both intervention and implementation trials [10]. For clinical or public health interventions, pilot and feasibility studies may serve to identify potential refinements to the intervention, address uncertainties around the feasibility of intervention trial methods, or test preliminary effects of the intervention [10]. In implementation research, feasibility and pilot studies perform the same functions as those for intervention trials, however with a focus on developing or refining implementation strategies, refining research methods for an implementation intervention trial, or undertake preliminary testing of implementation strategies [14, 15]. Despite this, reviews of implementation studies appear to suggest that few full implementation randomised controlled trials have undertaken feasibility and pilot work in advance of a larger trial [16].

A range of publications provides guidance for the conduct of feasibility and pilot studies for conventional clinical or public health efficacy trials including Guidance for Exploratory Studies of complex public health interventions [17] and the Consolidated Standards of Reporting Trials (CONSORT 2010) for Pilot and Feasibility trials [18]. However, given the differences between implementation trials and conventional clinical or public health efficacy trials, the field of implementation science has identified the need for nuanced guidance [14–16, 19, 20]. Specifically, unlike traditional feasibility and pilot studies that may include the preliminary testing of interventions on individual clinical or public health outcomes, implementation feasibility and pilot studies that explore strategies to improve intervention implementation often require assessing changes across multiple levels including individuals (e.g. service providers or clinicians) and organisational systems [21]. Due to the complexity of influencing behaviour change, the role of feasibility and pilot studies of implementation may also extend to identifying potential causal mechanisms of change and facilitate an iterative process of refining intervention strategies and optimising their impact [16, 17]. In addition, where conventional clinical or public health efficacy trials are typically conducted under controlled conditions and directed mostly by researchers, implementation trials are more pragmatic [15]. As is the case for well conducted effectiveness trials, implementation trials often require partnerships with end-users and at times, the prioritisation of end-user needs over methods (e.g. random assignment) that seek to maximise internal validity [15, 22]. These factors pose additional challenges for implementation researchers and underscore the need for guidance on conducting feasibility and pilot implementation studies.

### Aim

Given the importance of feasibility and pilot studies in improving implementation strategies and the quality of full-scale trials of those implementation strategies, our aim is to provide practice guidance for those undertaking formative feasibility or pilot studies in the field of implementation science. Specifically, we seek to provide guidance pertaining to the three possible purposes of undertaking pilot and feasibility studies, namely (i) to inform implementation strategy development, (ii) to assess potential implementation strategy effects and (iii) to assess the feasibility of study methods.

## Method

A series of three facilitated group discussions were conducted with a group comprising of the 6 members from Canada, the U.S. and Australia (authors of the manuscript) that were mutually interested in the use of feasibility and pilot trials in implementation science. Members included international experts in implementation and behavioural science, public health and trial methods, and had considerable experience in conducting feasibility, pilot and/ or implementation trials. The group was responsible for developing the guidance document, including identification and synthesis of pertinent literature, and approving the final guidance.

To inform guidance development, a literature review was undertaken in electronic bibliographic databases and google, to identify and compile existing recommendations and guidelines for feasibility and pilot studies broadly. Through this process, we identified 30 such guidelines and recommendations relevant to our aim [2, 10, 14, 15, 17, 18, 23–45]. In addition, seminal methods and implementation science texts recommended by the group were examined. These included the CONSORT 2010 Statement: extension to randomised pilot and feasibility trials [18], the Medical Research Council’s framework for development and evaluation of randomised controlled trials for complex interventions to improve health [2], the National Institute of Health Research (NIHR) definitions [39] and the Quality Enhancement Research Initiative (QUERI) Implementation Guide [4]. A summary of feasibility and pilot study guidelines and recommendations, and that of seminal methods and implementation science texts, was compiled by two authors. This document served as the primary discussion document in meetings of the group. Additional targeted searches of the literature were undertaken in circumstances where the identified literature did not provide sufficient guidance. The manuscript was developed iteratively over 9 months via electronic circulation and comment by the group. Any differences in views between reviewers was discussed and resolved via consensus during scheduled international video-conference calls. All members of the group supported and approved the content of the final document.

The broad guidance provided is intended to be used as supplementary resources to existing seminal feasibility and pilot study resources. We used the definitions of feasibility and pilot studies as proposed by Eldridge and colleagues [10]. These definitions propose that any type of study relating to the preparation for a main study may be classified as a “feasibility study”, and that the term “pilot” study represents a subset of feasibility studies that specifically look at a design feature proposed for the main trial, whether in part of in full, that is being conducted on a smaller scale [10]. In addition, when referring to pilot studies, unless explicitly stated otherwise, we will primarily focus on pilot trials using a randomised design. We focus on randomised trials as such designs are the most common trial design in implementation research, and randomised designs may provide the most robust estimates of the potential effect of implementation strategies [46]. Those undertaking pilot studies that employ non-randomised designs need to interpret the guidance provided in this context. We acknowledge, however, that using randomised designs can prove particularly challenging in the field of implementation science, where research is often undertaken in real-world contexts with pragmatic constraints.

We used the effectiveness-implementation hybrid trial design typology proposed by Curran and colleagues as the framework for conceptualising the application of feasibility testing of implementation interventions [47]. The typology makes an explicit distinction between the purpose and methods of implementation and conventional clinical (or public health efficacy) trials. Specifically, the first two of the three hybrid designs may be relevant for implementation feasibility or pilot studies. Hybrid Type 1 trials are those designed to test the effectiveness of an intervention on clinical or public health outcomes (primary aim) while conducting a feasibility or pilot study for future implementation via observing and gathering information regarding implementation in a real-world setting/situation (secondary aim) [47]. Hybrid Type 2 trials involve the simultaneous testing of both the clinical intervention and the testing or feasibility of a formed implementation intervention/strategy as co-primary aims. For this design, “testing” is inclusive of pilot studies with an outcome measure and related hypothesis [47]. Hybrid Type 3 trials are definitive implementation trials designed to test the effectiveness of an implementation strategy whilst also collecting secondary outcome data on clinical or public health outcomes on a population of interest [47]. As the implementation aim of the trial is a definitively powered trial, it was not considered relevant to the conduct of feasibility and pilot studies in the field and will not be discussed.

Embedding of feasibility and pilot studies within Type 1 and Type 2 effectiveness-implementation hybrid trials has been recommended as an efficient way to increase the availability of information and evidence to accelerate the field of implementation science and the development and testing of implementation strategies [4]. However, implementation feasibility and pilot studies are also undertaken as stand-alone exploratory studies and do not include effectiveness measures in terms of the patient or public health outcomes. As such, in addition to discussing feasibility and pilot trials embedded in hybrid trial designs, we will also refer to stand-alone implementation feasibility and pilot studies.

## Guidance

An overview of guidance (aims, design, measures, sample size and power, progression criteria and reporting) for feasibility and pilot implementation studies can be found in Table 1.

**Table 1:** Summary of considerations for implementation feasibility and pilot studies [18, 48–50]

	Potential objectives*		
	Implementation strategy development	Implementation effectiveness	Implementation trial methods
Aims	To assess or describe contextual and environmental factors in order to inform the development of an implementation strategy.	To test the potential impact of an implementation strategy.	To assess or describe the feasibility, utility, acceptability or quality of trial methods.
Design	Formative, non-comparative designs. Focus usually on qualitative or mixed methods approaches. Stand-alone study or as part of a Hybrid Type 1 design.	Summative and formative. Focus on comparative quantitative designs such as randomised or cluster randomised designs Stand-alone study or as part of a Hybrid Type 2 design.	Summative and formative Focus may be quantitative, qualitative or mixed methods approaches. Stand-alone study or as part of a Hybrid type 1 or 2 trial.
Measures	Measures informing design or development of implementation strategy such as context, acceptability, adaptability, feasibility, compatibility, cost, culture, dose, complexity and self-efficacy.	Measures of impact of implementation such as adoption, reach, fidelity and sustainability along with measures as per non-pilot implementation studies.	Measures informing implementation trial methods including the feasibility, acceptability or quality of data collection procedures, survey items, tools, or data management strategies.
Sample size and power	Justification of sample size based on achieving estimates of sufficient precision.	Justification of sample size based on achieving estimates of sufficient precision to inform trial progression (using progression criteria).	Justification of sample size based on achieving estimates of sufficient precision—which may or may not be linked to progression criteria.
Progression Criteria	Not required given such studies are formative.	Progression criteria set a priori based estimates of potential effects. Progression may be considered in conjunction with measures feasibility, acceptability or quality of methods (or other factors).	Progression criteria may be set a priori in summative pilot trials assessing trial methods. Progression may be considered in conjunction with estimates of potential trial effects (or other factors).
Reporting	Draw on relevant aspects of CONSORT extension for randomised pilot and feasibility trials, STaRi and reporting guidelines specific to the research design (e.g. STROBE)	Draw on upon existing reporting standards such as CONSORT extension for randomised pilot and feasibility trials, STaRi guidelines and TIDieR.	Draw on upon existing reporting standards such as CONSORT extension for randomised pilot and feasibility trials, STaRi guidelines and reporting guidelines specific to the research design.

*Implementation feasibility or pilot studies may have multiple objectives, for example, pilot implementation studies embedded in Hybrid Type 2 trials may also aim to inform implementation strategy development

*CONSORT* Consolidated Standards of Reporting Trials, *STaRi* Standards for Reporting Implementation Studies, *STROBE* Strengthening the Reporting of Observational Studies in Epidemiology, *TIDieR* Template for Intervention Description and Replication

### Purpose (aims)

The primary objective of hybrid type 1 trial is to assess the effectiveness of a clinical or public health intervention (rather than an implementation strategy) on the patient or population health outcomes [47]. Implementation strategies employed in these trials are often designed to maximise the likelihood of an intervention effect [51], and may not be intended to represent the strategy that would (or could feasibly), be used to support implementation in more “real world” contexts. Specific aims of implementation feasibility or pilot studies undertaken as part of Hybrid Type 1 trials are therefore formative and descriptive as the implementation strategy has not been fully formed nor will be tested. Thus, the purpose of a Hybrid Type 1 feasibility study is generally to inform the development or refinement of the implementation strategy rather than to test potential effects or mechanisms [22, 47]. An example of a Hybrid Type 1 trial by Cabassa and colleagues is provided in Additional file 1 [52].

In Hybrid Type 2 trial designs, there is a dual purpose to test: (i) the clinical or public health effectiveness of the intervention on clinical or public health outcomes (e.g. measure of disease or health behaviour) and (ii) test or measure the impact of the implementation strategy on implementation outcomes (e.g. adoption of health policy in a community setting) [53]. However, testing the implementation strategy on implementation outcomes may be a secondary aim in these trials and positioned as a pilot [22]. In Hybrid Type 2 trial designs, the implementation strategy is more developed than in Hybrid Type 1 trials, resembling that intended for future testing in a definitive implementation randomised controlled trial. The dual testing of the evidence-based intervention and implementation interventions or strategies in Hybrid Type 2 trial designs allows for direct assessment of potential effects of an implementation strategy and exploration of components of the strategy to further refine logic models. Additionally, such trials allow for assessments of the feasibility, utility, acceptability or quality of research methods for use in a planned definitive trial. An example of a Hybrid Type 2 trial design by Barnes and colleagues [54] is included in Additional file 2.

Non-hybrid pilot implementation studies are undertaken in the absence of a broader effectiveness trial. Such studies typically occur when the effectiveness of a clinical or public health intervention is well established, but robust strategies to promote its broader uptake and integration into clinical or public health services remain untested [15]. In these situations, implementation pilot studies may test or explore specific trial methods for a future definitive randomised implementation trial. Similarly, a pilot implementation study may also be undertaken in a way that provides a more rigorous formative evaluation of hypothesised implementation strategy mechanisms [55], or potential impact of implementation strategies [56], using similar approaches to that employed in Hybrid Type 2 trials. Examples of potential aims for feasibility and pilot studies are outlined in Table 2.

**Table 2:** Potential aims of implementation feasibility studies and pilot studies

Implementation study design	Implementation strategy development	Preliminary implementation effectiveness	Implementation trial methods
**Non-pilot feasibility studies (for example as part of Hybrid Type 1 trials)**	Explore implementation strategies (e.g., what supports are required and how would they be best delivered in order for service providers to undertake the intervention as part of routine practice?) [15]. Describe barriers and enablers to implementation strategies (e.g., What factors may influence the uptake of implementation strategies?) [56]. Describe acceptability, feasibility and/or appropriateness of implementation strategies [56].	Usually not part of non-pilot feasibility studies or Hybrid Type 1 designs.	Describe any organisational/contextual factors that may influence future implementation trial methods, such as recruitment, retention, data collection procedures and number (sample size) and type (diversity) of organisations required for a future implementation trial [15].
**Pilot studies (for example as part of Hybrid Type 2 trials)**	Assess barriers and enablers to delivery of the implementation strategies (e.g., What are factors that influenced the use of intervention? What are the factors that influenced fidelity to implementation?) [56]. Describe acceptability, feasibility and/or appropriateness of implementation strategies [56]. Establish preliminary evidence of strategy mechanisms (e.g., To determine if there is preliminary evidence that the hypothesised mechanism is responsible for the effect of the implementation strategy) [55].	To test potential effects of the implementation intervention (e.g., through measures such as adoption, fidelity, reach) [56].	To assess methods such as recruitment, retention, data collection tools and procedures, number (sample size) of organisations and type (diversity) of organisations, in order to inform a future implementation trial [17].

### Design

For implementation feasibility or pilot studies, as is the case for these types of studies in general, the selection of research design should be guided by the specific research question that the study is seeking to address [57]. Although almost any study design may be used, researchers should review the merits and potential threats to internal and external validity to help guide the selection of research design for feasibility/pilot testing [15].

As Hybrid Type 1 trials are primarily concerned with testing the effectiveness of an intervention (rather than implementation strategy), the research design will typically employ power calculations and randomisation procedures at the health outcome level to measure the effect on behaviour, symptoms, functional and/or other clinical or public health outcomes. Hybrid Type 1 feasibility studies may employ a variety of designs usually nested within the experimental group (those receiving the intervention and any form of an implementation support strategy) of the broader efficacy trial [47]. Consistent with the aims of Hybrid Type 1 feasibility and pilot studies, the research designs employed are likely to be non-comparative. Cross-sectional surveys, interviews or document review, qualitative research or mix methods approaches may be used to assess implementation contextual factors, such as barriers and enablers to implementation and/or the acceptability, perceived feasibility or utility of implementation strategies or research methods [47].

Pilot implementation studies as part of Hybrid Type 2 designs can make use of the comparative design of the broader effectiveness trial to examine the potential effects of the implementation strategy [47] and more robustly assess the implementation mechanisms, determinants and influence of broader contextual factors [53]. In this trial type, mixed method and qualitative methods may complement the findings of between group (implementation strategy arm versus comparison) quantitative comparisons, enable triangulation and provide more comprehensive evidence to inform implementation strategy development and assessment. Stand-alone implementation feasibility and pilot implementation studies are free from the constraints and opportunities of research embedded in broader effectiveness trials. As such, research can be designed in a way that best addresses the explicit implementation objectives of the study. Specifically, non-hybrid pilot studies can maximise the applicability of study findings for future definitive trials by employing methods to directly test trial methods such as recruitment or retention strategies [17], enabling estimates of implementation strategies effects [56] or capturing data to explicitly test logic models or strategy mechanisms.

### Measures

The selection of outcome measures should be linked directly to the objectives of the feasibility or pilot study. Where appropriate, measures should be objective or have suitable psychometric properties, such as evidence of reliability and validity [58, 59]. Public health evaluation frameworks often guide the choice of outcome measure in feasibility and pilot implementation work and include RE_AIM [60], PRECEDE_PROCEED [61], Proctor and colleagues framework on outcomes for implementation research [62] and more recently, the “Implementation Mapping” framework [63]. Recent work by McKay and colleagues suggests a minimum data set of implementation outcomes that includes measures of adoption, reach, dose, fidelity and sustainability [46]. We discuss selected measures below and provide a summary in Table 3 [46]. Such measures could be assessed using quantitative or qualitative or mixed methods [46].

**Table 3:** Illustrations of implementation strategy measures for use in feasibility and pilot implementation studies^a^ [15, 17, 44, 46, 60, 62, 65, 68, 70–72]

Purpose	Measures	Examples of potential implementation feasibility and pilot study measures (including as part of Hybrid Type 1 trials)	Examples of potential implementation pilot trial measures (including as part of Hybrid Type 2 trials)
**To assess potential implementation strategy effects**	**Adoption:** the proportion and representativeness of settings and staff that adopt the innovation [60]	Assessment of implementation strategy effects are not typically part of non-pilot feasibility or Type 1 Hybrid trials ^b^	Percent and type of service providers utilising the intervention Percent and type of support system teams/ staff members undertaking the implementation strategies [60]
	**Fidelity (adherence):** the degree to which the innovation is implemented as intended by developers [70]		Measures such as content, frequency, duration, and coverage as prescribed by its designers [70]. Number and type of adaptations made to implementation strategies including information on how and why [68].
	**Reach (penetration)** Participation rate in the innovation by the intended audience [62]		The proportion of support systems staffs’ participation in the delivery of the implementation strategy [46].
	**Sustainability (maintenance):** continuation or maintenance of the innovation’s desired changes [62]		Uptake of implementation strategies by support systems continued at a specified time(s) post the initial intervention [15].
**To inform the design or development of the implementation strategy (determinants)**	**Adaptability:** the degree to which the innovation can be adapted to meet local needs [65]	Organisations’ view of the flexibility required for future implementation strategies.	To what extent did support systems find they could tailor or adapt implementation strategies (whilst maintaining core components) [46]
	**Acceptability:** Service providers or support system’s satisfaction with the innovation [62]	If service providers and / or support systems approve of proposed future implementation strategies (such as content or proposed delivery)	If service providers or support systems found the implementation components agreeable, for example in terms of content or delivery [46, 62]
	**Feasibility:** actual fit or suitability of the innovation for everyday use [62]	If service providers and/ or support systems staff agree with the suitability of proposed future implementation strategies	If service providers and support systems staff agree that the implementation strategies were able to be successfully undertaken [46].
	**Compatibility (appropriateness):** perceived fit of the innovation with organisation’s values, mission, priorities [71]	If support systems agree that any future proposed implementation strategy is in line with organisational priorities	If support systems agree that the implementation strategies are in line with organisational priorities [46].
	**Dose (satisfaction)** Satisfaction with the dose of the innovation received [72]	Implementation strategies typically not delivered in non-pilot feasibility or Hybrid Type 1 trials	If support systems are satisfied with the amount of support and resources received as part of implementation strategies [46].
	**Complexity** Perception of difficulty of implementation/ number of components of the innovation [65]	If service providers or support systems perceive difficulty carrying out proposed future implementation strategies For example, due to duration, scope, intricacy and disruptiveness.	If support systems found the implementation strategies difficult to undertake. For example, due to duration, scope, intricacy and disruptiveness [65].
	**Context** Political, economic or social influences on implementation of the innovation [46]	If any organisational political, economic or social factors would influence the uptake of future implementation strategies.	If any organisational political, economic or social factors did influence the uptake of implementation strategies [46].
	**Culture** Organisational norms, values or basic assumptions influencing implementation of the innovation [65]	If setting or organisational values, norms and assumptions influence may influence the uptake of future implementation strategies. For example, work structures and behaviours.	If setting or organisational values, norms influenced the uptake of the implementation strategies For example, work structures and behaviours [65].
	**Self-efficacy** Self-belief in the ability to execute goals of the innovation [46]	If support systems staff believe in their capacity (e.g. knowledge and skills) to complete any future implementation strategies	If support systems staff agree they had the capacity (e.g. knowledge and skills) to undertake implementation strategies [46].
	**Cost** Measures of the cost or relative cost of implementation of the innovation [62]	Collection of data to help project cost of future implementation.	Cost to deliver the innovation [62].
**To assess feasibility of trial methods**	**Feasibility of future trial design to conduct a full trial** [17]	If the organisation and/or support systems perceive proposed future implementation trial design components to be feasible For example, feasibility of proposed recruitment methods, acceptability of data collection procedures and tools etc.	If the pilot trial design and methods are feasible to replicate as part of a larger implementation trial. For example, the feasibility of recruitment methods, site and participant retention, implementation data collection procedures and tools etc. [17, 44]

^a^Table populated based on measures and terminology reported in McKay et al. [46]

^b^These factors could be assessed in evidence-based interventions in Hybrid Type 1 trials

Service providers: clinicians, primary health care providers, or other providers of health-related programs who deliver the evidence-based intervention [46]

Support systems: the resource team at the organisational or settings level who support or deliver implementation strategies [46]

Innovation: refers to the intervention in its entirety and is used to encompass the inclusion of measures specific to the delivery of implementation content and, if applicable, the delivery of the intervention as would be the case in hybrid trial designs

#### Measures to assess potential implementation strategy effects

In addition to assessing the effects of an intervention on individual clinical or public health outcomes, Hybrid Type 2 trials (and some non-hybrid pilot studies) are interested in measures of the potential effects of an implementation strategy on desired organisational or clinician practice change such as adherence to a guideline, process, clinical standard or delivery of a program [62]. A range of potential outcomes that could be used to assess implementation strategy effects has been identified, including measures of adoption, reach, fidelity and sustainability [46]. These outcomes are described in Table 2, including definitions and examples of how they may be applied to the implementation component of innovation being piloted. Standardised tools to assess these outcomes are often unavailable due to the unique nature of interventions being implemented and the variable (and changing) implementation context in which the research is undertaken [64]. Researchers may collect outcome data for these measures as part of environmental observations, self-completed checklists or administrative records, audio recording of client sessions or other methods suited to their study and context [62]. The limitations of such methods, however, need to be considered.

#### Measures to inform the design or development of the implementation strategy

Measures informing the design or development of the implementation strategy are potentially part of all types of feasibility and pilot implementation studies. An understanding of the determinants of implementation is critical to implementation strategy development. A range of theoretical determinant frameworks have been published which describe factors that may influence intervention implementation [65], and systematic reviews have been undertaken describing the psychometric properties of many of these measures [64, 66]. McKay and colleagues have also identified a priority set of determinants for implementation trials that could be considered for use in implementation feasibility and pilot studies, including measures of context, acceptability, adaptability, feasibility, compatibility, cost, culture, dose, complexity and self-efficacy [46]. These determinants are described in Table 3, including definitions and how such measures may be applied to an implementation feasibility or pilot study. Researchers should consider, however, the application of such measures to assess both the intervention that is being implemented (as in a conventional intervention feasibility and pilot study) and the strategy that is being employed to facilitate its implementation, given the importance of the interaction between these factors and implementation success [46]. Examples of the potential application of measures to both the intervention and its implementation strategies have been outlined elsewhere [46]. Although a range of quantitative tools could be used to measure such determinants [58, 66], qualitative or mixed methods are generally recommended given the capacity of qualitative measures to provide depth to the interpretation of such evaluations [40].

**Fig. 2:**
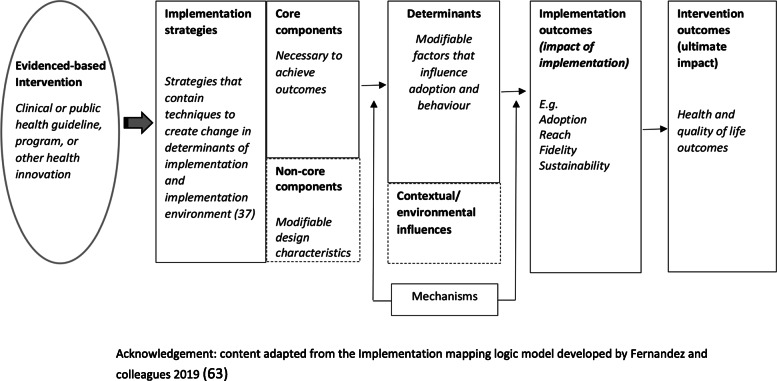
Example of components of an Implementation logic model

Measures of potential implementation determinants may be included to build or enhance logic models (Hybrid Type 1 and 2 feasibility and pilot studies) and explore implementation strategy mechanisms (Hybrid Type 2 pilot studies and non-hybrid pilot studies) [67]. If exploring strategy mechanisms, a hypothesized logic model underpinning the implementation strategy should be articulated including strategy-mechanism linkages, which are required to guide the measurement of key determinants [55, 63]. An important determinant which can complicate logic model specification and measurement is the process of adaptation—modifications to the intervention or its delivery (implementation), through the input of service providers or implementers [68]. Logic models should specify components of implementation strategies thought to be “core” to their effects and those which are thought to be “non-core” where adaptation may occur without adversely impacting on effects. Stirman and colleagues propose a method for assessing adaptations that could be considered for use in pilot and feasibility studies of implementation trials [69]. Figure 2 provides an example of some of the implementation logic model components that may be developed or refined as part of feasibility or pilot studies of implementation [15, 63].

#### Measures to assess the feasibility of study methods

Measures of implementation feasibility and pilot study methods are similar to those of conventional studies for clinical or public health interventions. For example, standard measures of study participation and thresholds for study attrition (e.g. >20%) rates [73] can be employed in implementation studies [67]. Previous studies have also surveyed study data collectors to assess the success of blinding strategies [74]. Researchers may also consider assessing participation or adherence to implementation data collection procedures, the comprehension of survey items, data management strategies or other measures of feasibility of study methods [15].

### Pilot study sample size and power

In effectiveness trials, power calculations and sample size decisions are primarily based on the detection of a clinically meaningful difference in measures of the effects of the intervention on the patient or public health outcomes such as behaviour, disease, symptomatology or functional outcomes [24]. In this context, the available study sample for implementation measures included in Hybrid Type 1 or 2 feasibility and pilot studies may be constrained by the sample and power calculations of the broader effectiveness trial in which they are embedded [47]. Nonetheless, a justification for the anticipated sample size for all implementation feasibility or pilot studies (hybrid or stand-alone) is recommended [18], to ensure that implementation measures and outcomes achieve sufficient estimates of precision to be useful. For Hybrid type 2 and relevant stand-alone implementation pilot studies, sample size calculations for implementation outcomes should seek to achieve adequate estimates of precision deemed sufficient to inform progression to a fully powered trial [18].

### Progression criteria

Stating progression criteria when reporting feasibility and pilot studies is recommended as part of the CONSORT 2010 extension to randomised pilot and feasibility trials guidelines [18]. Generally, it is recommended that progression criteria should be set a priori and be specific to the feasibility measures, components and/or outcomes assessed in the study [18]. While little guidance is available, ideas around suitable progression criteria include assessment of uncertainties around feasibility, meeting recruitment targets, cost-effectiveness and refining causal hypotheses to be tested in future trials [17]. When developing progression criteria, the use of guidelines is suggested rather than strict thresholds [18], in order to allow for appropriate interpretation and exploration of potential solutions, for example, the use of a traffic light system with varying levels of acceptability [17, 24]. For example, Thabane and colleagues recommend that, in general, the outcome of a pilot study can be one of the following: (i) stop—main study not feasible (red); (ii) continue, but modify protocol—feasible with modifications (yellow); (iii) continue without modifications, but monitor closely—feasible with close monitoring and (iv) continue without modifications (green) (44)p5.

As the goal of Hybrid Type 1 implementation component is usually formative, it may not be necessary to set additional progression criteria in terms of the implementation outcomes and measures examined. As Hybrid Type 2 trials test an intervention and can pilot an implementation strategy, criteria for these and non-hybrid pilot studies may set progression criteria based on evidence of potential effects but may also consider the feasibility of trial methods, service provider, organisational or patient (or community) acceptability, fit with organisational systems and cost-effectiveness [17]. In many instances, the progression of implementation pilot studies will often require the input and agreement of stakeholders [27]. As such, the establishment of progression criteria and the interpretation of pilot and feasibility study findings in the context of such criteria require stakeholder input [27].

### Reporting suggestions

As formal reporting guidelines do not exist for hybrid trial designs, we would recommend that feasibility and pilot studies as part of hybrid designs draw upon best practice recommendations from relevant reporting standards such as the CONSORT extension for randomised pilot and feasibility trials, the Standards for Reporting Implementation Studies (STaRI) guidelines and the Template for Intervention Description and Replication (TIDieR) guide as well as any other design relevant reporting standards [48, 50, 75]. These, and further reporting guidelines, specific to the particular research design chosen, can be accessed as part of the EQUATOR (Enhancing the QUAility and Transparency Of health Research) network—a repository for reporting guidance [76]. In addition, researchers should specify the type of implementation feasibility or pilot study being undertaken using accepted definitions. If applicable, specification and justification behind the choice of hybrid trial design should also be stated. In line with existing recommendations for reporting of implementation trials generally, reporting on the referent of outcomes (e.g. specifying if the measure in relation to the specific intervention or the implementation strategy) [62], is also particularly pertinent when reporting hybrid trial designs.

## Conclusions

Concerns are often raised regarding the quality of implementation trials and their capacity to contribute to the collective evidence base [3]. Although there have been many recent developments in the standardisation of guidance for implementation trials, information on the conduct of feasibility and pilot studies for implementation interventions remains limited, potentially contributing to a lack of exploratory work in this area and a limited evidence base to inform effective implementation intervention design and conduct [15]. To address this, we synthesised the existing literature and provide commentary and guidance for the conduct of implementation feasibility and pilot studies. To our knowledge, this work is the first to do so and is an important first step to the development of standardised guidelines for implementation-related feasibility and pilot studies.

## Supplementary information

**Additional file 1.** Example of a Hybrid Type 1 trial. Summary of publication by Cabassa et al.

**Additional file 2.** Example of a Hybrid Type 2 trial. Summary of publication by Barnes et al.

## Data Availability

Not applicable.

## Abbreviations

RCT: Randomised controlled trial
CONSORT: Consolidated Standards of Reporting Trials
EQUATOR: Enhancing the QUAility and Transparency Of health Research
STaRI: Standards for Reporting Implementation Studies
STROBE: Strengthening the Reporting of Observational Studies in Epidemiology
TIDieR: Template for Intervention Description and Replication
NIHR: National Institute of Health Research
QUERI: Quality Enhancement Research Initiative
