# Supplementary material for: Guidance for conducting feasibility and pilot studies for implementation trials
Source: Pilot Feasibility Stud. 2020 Oct 31;6:167. doi: 10.1186/s40814-020-00634-w (PMC7603668; doi:10.1186/s40814-020-00634-w)
Supplement: Supplementary file 1 — Additional file 1. Example of a Hybrid Type 1 trial. Summary of publication by Cabassa et al. [file 40814_2020_634_MOESM1_ESM.docx]

**Additional file 1**

***Example of a Hybrid Type 1 trial***

**Purpose:** Cabassa and colleagues sought to test the effectiveness of a peer-led weight management intervention for mental health clients accessing supportive housing agencies. In addition, as part of the Hybrid type 1 design, researchers undertook a feasibility study to examine implementation factors and processes that may influence the use of the weight management intervention in routine practice.

**Design:** The effectiveness component of the trial was a randomised control trial of a 12- month group healthy lifestyle intervention for weight loss versus usual care. A mixed methods design, including focus groups, interviews and surveys, was utilised for the feasibility study.

**Sample:** The effectiveness study aimed to recruit 300 clients from three supportive housing agencies sites in order to detect a difference in weight loss of ≥5% (alpha 0.05, minimum 80% power). For the feasibility study, staff at various levels of the organisation (e.g., service providers and directors) were recruited from each site, with directors used as key informants to nominate appropriate program managers to also form part of the sample.

**Outcomes:** The primary outcome measure for the effectiveness trial was percent weight loss at 6, 12 and 18 months. Outcomes for the feasibility trial include the influence of outer/inner context of the study sites, and the systems, organisational and staff level factors that may hinder or facilitate implementation.

**Measures (implementation):** Directors and program managers undertook semi-structured qualitative interviews and self-administered surveys and focus groups were undertaken with direct service providers and peer specialists. Surveys included measures of staff characteristics, organisational culture, the organisation’s recovery orientation and attitudes towards evidence-based practice. Focus groups explored the climate and culture of the organisation, views on evidence-based practice, views on the program and the service providers’ experience of delivering the program.

**Results informing implementation**: Quantitative data were reported at the level of the organisation and qualitative data was then integrated with quantitative findings. A “heuristic model” was then developed to inform the development of future implementation strategies.(Cabassa et al. 2015) The study found that implementation decisions were influenced by a combination of funding concerns, external regulations and pressures, fit with the organization’s mission, leadership support, concerns about administrative burdens, and the importance of buy-in from both staff and clients. (Cabassa et al. 2019)

Cabassa LJ, Stefancic A, O'Hara K, El-Bassel N, Lewis-Fernández R, Luchsinger JA, et al. Peer-led healthy lifestyle program in supportive housing: study protocol for a randomized controlled trial. Trials. 2015;16:388.

Cabassa J, Stefancic A. Context before implementation: a qualitative study of decision. TBM. 2019;9:217–226.

doi: 10.1093/tbm/iby0342019;

makers’ views of a peer-led healthy lifestyle intervention

for people with serious mental illness in supportive

housing
