# Supplementary material for: Guidance for conducting feasibility and pilot studies for implementation trials
Source: Pilot Feasibility Stud. 2020 Oct 31;6:167. doi: 10.1186/s40814-020-00634-w (PMC7603668; doi:10.1186/s40814-020-00634-w)
Supplement: Supplementary file 2 — Additional file 2. Example of a Hybrid Type 2 trial. Summary of publication by Barnes et al. [file 40814_2020_634_MOESM2_ESM.docx]

**Additional file 2**

***Example of a Hybrid Type 2 trial inclusive of a pilot implementation trial***

**Purpose:** Barnes and colleagues describe the protocol for a Hybrid Type 2 trial. The primary aim is to determine the effectiveness the intervention on fruit and vegetable intake of children in care. The implementation component of the trial aims to pilot test the potential effects of the implementation strategies on uptake of healthy eating policy and practices that promote fruit and vegetable consumption.

**Design:** A cluster randomised controlled trial of 22 services allocated to either the intervention or usual care. The intervention consists of a web-based program to action plan and support change, appointment of a centre champion, support from health promotion officers, training and resources.

**Sample:** The trial aims to recruit 440 children from 22 services in order to detect a mean difference of 0.3 serves in fruit and vegetables (alpha 0.05, ICC 0.1, 80% power). Appropriate staff from each intervention service will be identified to take part in surveys to measure determinants of implementation.

**Outcomes:** The primary outcome measure of the intervention effectiveness is serves of fruit and vegetables in children lunchboxes. Outcomes for the pilot of implementation strategy include uptake of healthy eating policy and practices that support fruit and vegetable consumption and the acceptability, feasibility, appropriateness, and contextual factors influencing implementation.

**Measures:** Fruit and vegetable consumption will be assessed via weighing and photography of children’s lunchboxes pre and post meals at baseline and follow-up. Measures of the potential effects the implementation strategy will be assessed via observations of the centre nutrition environment and collection of service internal records. Fidelity of the implementation strategy delivery will be assessed via measures including childcare service use of supportive resources including use of the web-based program, evidence of appointment of a centre champion, completion rates of training and frequency and duration of researcher support accessed throughout the trial. Acceptability, feasibility, appropriateness will be measured via telephone surveys of centre staff in the intervention group, post intervention. Damschroder’s Consolidated Framework for Implementation Research (CFIR) was used to guide measures of contextual factors influencing implementation (ref).

**Progression criteria:** Progression criteria will include consideration of estimates of effect size on child dietary outcomes, alongside pre-specified measures of feasibility and acceptability of the intervention and implementation strategies. Consensus on the interpretation of data will be reached with core research team members in conjunction with a partner from the intervention setting.

Barnes C, Grady A, Nathan N, Wolfenden L, Pond N, McFauden T, Ward D, Vaughn A, Yoong S. A cluster randomised controlled trial of a web-based intervention to increase child intake of fruit and vegetables within childcare centres: a study protocol. Unpublished.
